# Analytical validity of DecisionDx-SCC, a gene expression profile test to identify risk of metastasis in cutaneous squamous cell carcinoma (SCC) patients

**DOI:** 10.1186/s13000-022-01211-w

**Published:** 2022-02-25

**Authors:** Sherri Borman, Jeff Wilkinson, Lauren Meldi-Sholl, Clare Johnson, Kelsey Carter, Kyle R. Covington, Alison L. Fitzgerald, Sarah J. Kurley, Aaron S. Farberg, Matthew S. Goldberg, Federico A. Monzon, Kristen Oelschlager, Robert W. Cook

**Affiliations:** 1 Castle Biosciences, Inc, Phoenix, AZ USA; 2 Castle Biosciences, Inc, 505 S. Friendswood Dr., Ste 400, Friendswood, TX 77546 USA; 3 Baylor Scott & White Health System, Dallas, TX USA; 4 Icahn School of Medicine at Mount Sinai, New York, NY USA

**Keywords:** Gene expression profiling, DecisionDx-SCC, Cutaneous squamous cell carcinoma, Metastasis, Analytic validity

## Abstract

**Background:**

To improve identification of patients with cutaneous squamous cell carcinoma (SCC) at high risk for metastatic disease, the DecisionDx-SCC assay, a prognostic 40-gene expression profile (40-GEP) test, was developed and validated. The 40-GEP assay utilizes RT-PCR gene expression analysis on primary tumor biopsy tissue to evaluate the expression of 34 signature gene targets and 6 normalization genes. The test provides classifications of low risk (Class 1), moderate risk (Class 2A), and high risk (Class 2B) of metastasis within 3 years of diagnosis. The primary objective of this study was to validate the analytical performance of the 40-gene expression signature.

**Methods:**

The repeatability and reproducibility of the 40-GEP test was evaluated by performance of inter-assay, intra-assay, and inter-operator precision experiments along with monitoring the reliability of sample and reagent stability for class call concordance. The technical performance of clinical orders from September 2020 through July 2021 for the 40-GEP test was assessed.

**Results:**

Patient hematoxylin and eosin (H&E) stained slides were reviewed by a board-certified pathologist to assess minimum acceptable tumor content. Class specific controls (Class 1 and Class 2B) were evaluated with Levey-Jennings analysis and demonstrated consistent and reproducible results. Inter-assay, inter-operator and intra-assay concordance were all ≥90%, with short-term and long-term RNA stability also meeting minimum concordance requirements. Of the 2586 orders received, 93.5% remained eligible for testing, with 97.1% of all tested samples demonstrating actionable class call results.

**Conclusion:**

DecisionDx-SCC demonstrates a high degree of analytical precision, yielding high concordance rates across multiple performance experiments, along with exhibiting robust technical reliability on clinical samples.

## Background

Although prognosis of cutaneous squamous cell carcinoma (SCC) is generally favorable, an estimated 6% of the greater than 1,000,000 cases diagnosed annually develop regional or distant metastasis, with approximately 2% dying from this disease [1–3]. With the striking increase in incidence over the past 30 years [4], SCC continues to be an increasing burden on the healthcare system. The subset of SCC patients that carry an elevated risk of metastasis and death are termed ‘high risk.’ Current National Comprehensive Cancer Network (NCCN) guidelines [5] recommend treatment strategies based on broad criteria for identifying a high-risk SCC patient (those with a single clinicopathologic risk factor); unfortunately, this approach has led to inconsistent patient management decisions [6–8]. Tumor staging systems also are solely based on clinicopathologic factors for risk assessment, resulting in low positive predictive values (meaning many high-risk SCC patients do not progress to advanced disease) [9]. Therefore, improvement in the prediction of metastatic risk is crucial for early identification of patients at risk of poor outcomes and can greatly assist with the unmet clinical need of establishing more risk-appropriate patient management.

A prognostic 40-gene expression profile (40-GEP) test, clinically available as DecisionDx-SCC, has been developed and validated to show improved stratification of metastatic risk in high-risk SCC patients compared to current staging systems alone [9]. A neural network-based predictive algorithm based upon the normalized gene expression values of 34 discriminant genes was used to predict classification of metastatic tumor biology risk, along with 6 additional genes chosen as controls [9], resulting in a 40-gene expression profile. The 40-GEP assay has been clinically validated and classifies SCC tumors into low (Class 1), moderate (Class 2A), and high (Class 2B) risk groups for regional or distant metastasis within 3 years of diagnosis. Clinical utility studies have also supported the integration of the 40-GEP test with standard guidelines for more refined, risk-appropriate SCC patient management [10–13].

This study outlines the analytical validation of the 40-GEP test, including reproducibility data (inter- and intra-assay reliability and inter-operator precision), repeatability, stability, and technical experience of clinical testing in accordance with published guidelines [14, 15]. The analysis was developed and performed in a CAP-accredited, New York State-Approved, CLIA-certified high throughput, molecular laboratory with high quality, standard operating procedures (SOPs).

## Methods

### Sample and clinical data collection

Samples were collected under a protocol that was reviewed and approved by the appropriate Institutional Review Board (IRB) for each contributing institution. Laboratory personnel were blinded to clinical truth outcomes (e.g., metastasis). The 40-GEP assay was designed for use with RNA obtained from formalin-fixed, paraffin embedded (FFPE) tissue from the biopsy of pathologist-diagnosed primary invasive SCC tumors having  one or more clinicopathologic risk factors. Ten 5 μm thick tissue sections were cut from FFPE blocks containing the biopsied tissue. The first slide was stained with hematoxylin and eosin (H&E) and sent to Castle Biosciences’ centralized laboratory along with the nine subsequent unstained slides.

### RT-PCR preparation

FFPE samples were processed as previously described [9, 16]. An area representative of the tumor was identified on the H&E slide by a dermatopathologist. The corresponding area was then manually macro-dissected from the unstained tissue slides and pooled into a single tube by trained, molecular technologists. The clinically allowable minimum for testing was ≥40% tumor nuclei [9, 16]. The RNA was extracted from the FFPE samples using QiaSymphony auto-nucleic acid extractors (Qiagen) and quantified. The isolated RNA from the samples was divided into separate tubes (to be run concurrently) then followed by reverse transcription to cDNA using a High-Capacity cDNA Reverse Transcription Kit (Applied Biosciences). After a 14-cycle pre-amplification with a custom OpenArray PreAmp pooled assay (ThermoFisher Scientific) and dilution, the OpenArray chip (Applied Biosystems) was loaded using the QuantStudio 12 K Flex Accufill system (Applied Biosystems) and high throughput qPCR was performed using the QuantStudio 12 K Flex PCR system (Applied Biosystems). Resulting qPCR data were normalized to the internal control genes, GAPDH, for data analysis. Positive and negative controls included in each OpenArray assay were the following: (−) DNA control, a no-template, water control, and (+) binary class controls chosen from previously run samples, (a Class 1 control sample and a Class 2 control sample). To establish the stability and consistent level of performance of the assay, all samples were compared to a qPCR DNase control to assess potential levels of DNA contamination. All assay runs and sample results underwent a multifactorial quality control (QC) review process, including assessment of the number of amplified probes within each sample. A specimen was reported as a multiple gene failure (MGF) if more than 3 target genes failed to amplify across the panel.

### Risk assignment

**Fig. 1:**
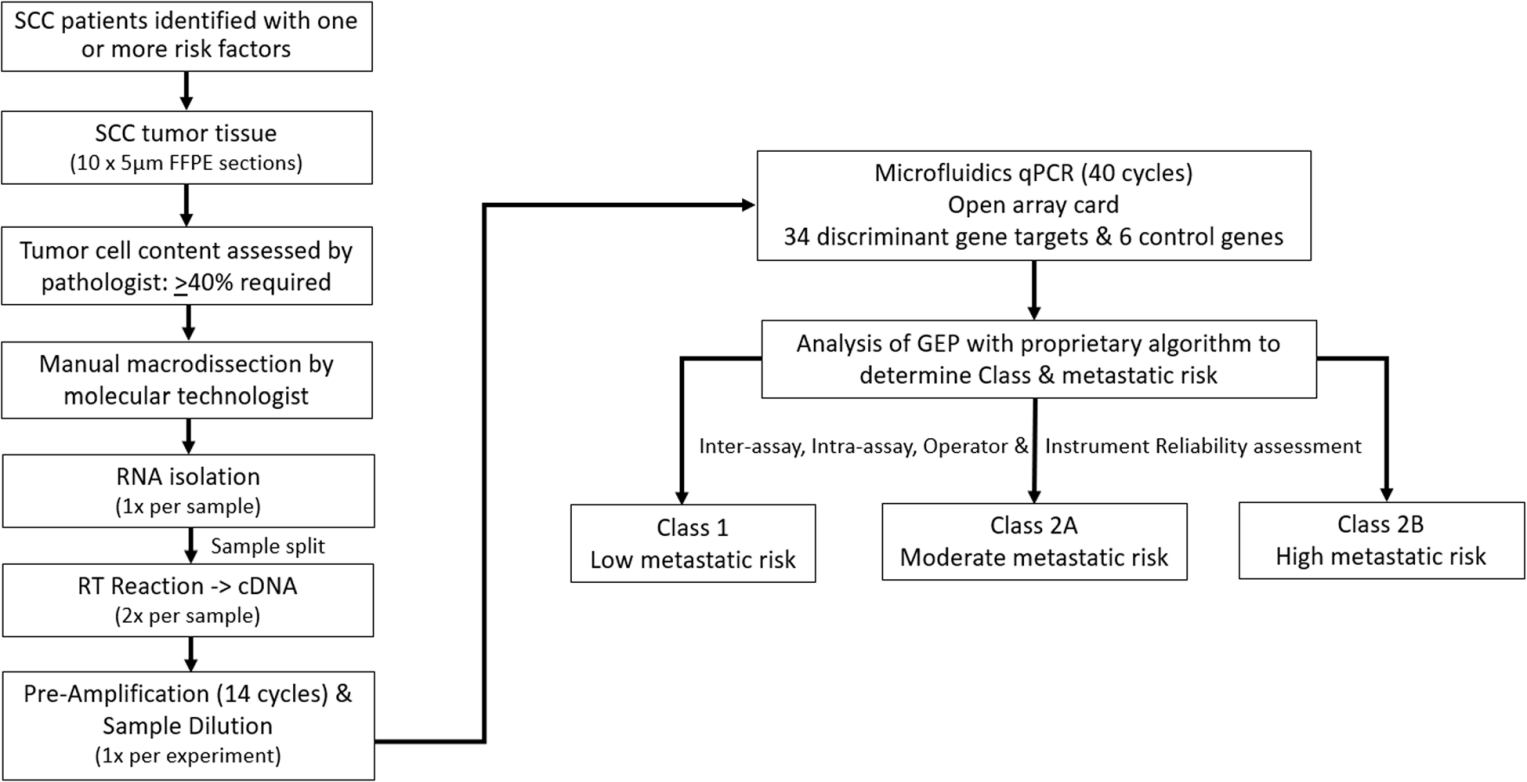
Workflow schematic of the DecisionDx-SCC test describing the steps in the performance of the test and presenting the corresponding reliability analyses

The expression of 6 “housekeeping” genes (BAG6, FXR1, KMT2C, KMT2D, MDM2, and MDM4) were utilized to normalize the expression of the 34 discriminant genes. The controls were chosen based on their robust expression and low variance [9]. Genes were arranged on the open array in triplicate to obtain an average Ct value for each gene. The Ct values for a given gene were then averaged for a final Ct value based on the geomean and normalized to the controls. The result was expressed as a ΔCt (the difference in expression level between the test gene and internal controls). The sample’s raw expression data was then subject to a proprietary algorithm comprised of two gene signatures (neural networks), both of which were established and locked following development with a training set of SCCs with known outcomes [9]. The algorithm returned two quantitative linear probability scores from 0 to 1.0 (Signature 1 and Signature 2). Each sample was run in duplicate, and the values of each signature were averaged, resulting in a qualitative classification of 3 possible risk groups: Class 1 (low risk), Class 2A (moderate risk), or Class 2B (high risk) tumor biology **(**Fig. 1**)**.

### Class call concordance determination

Analytic validity and reliability were reported as the qualitative concordance of class assignment and the correlation of quantitative probability score values. Class call concordance was evaluated in the three reported risk groups: Class 1, Class 2A, and Class 2B. To be deemed acceptable, the class calls between the original run and subsequent runs had to be ≥90% concordant.

## Results

### RNA input quantity and tumor content

**Fig. 2:**
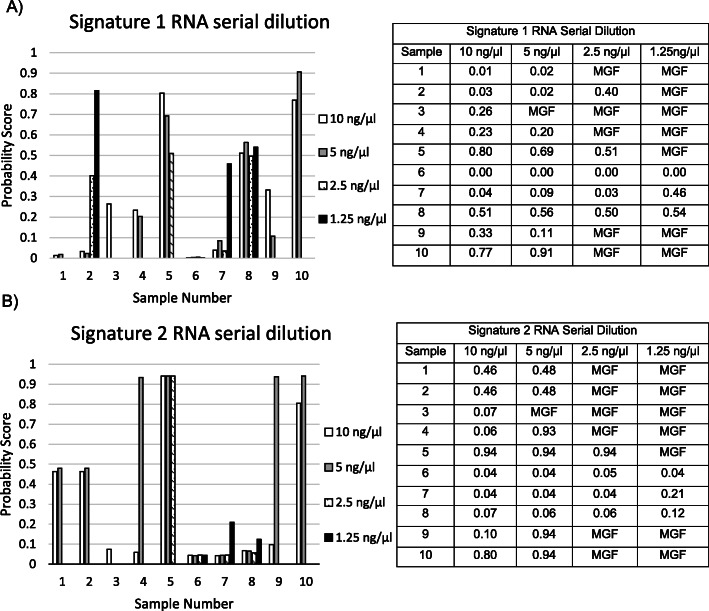
Determination of input RNA concentration using a serial dilution from 10 to 1.25 ng/μl, the probability scores of both A) Signature 1 and B) Signature 2 are shown for 10 FFPE samples. *MGF-multiple gene failure*

H&E-stained slides for each specimen tested were reviewed by a board-certified dermatopathologist to assess for both the amount of tumor tissue and the appropriate area to be macro-dissected. The minimum tumor content from a patient’s sample deemed acceptable was previously established at ≥40% [9, 16]. Following the dermatopathologist’s evaluation, samples were submitted to the laboratory where RNA from 10 samples with known concentrations were serially diluted from 10 ng/μl to 1.25 ng/μl **(**Fig. 2**)**. A dilution series was performed to establish minimum RNA inputs for the assay. On the basis of MGF rate, 5 ng/μl was determined to be the lower input limit for RNA concentration.

### Repeatability- assay robustness

**Fig. 3:**
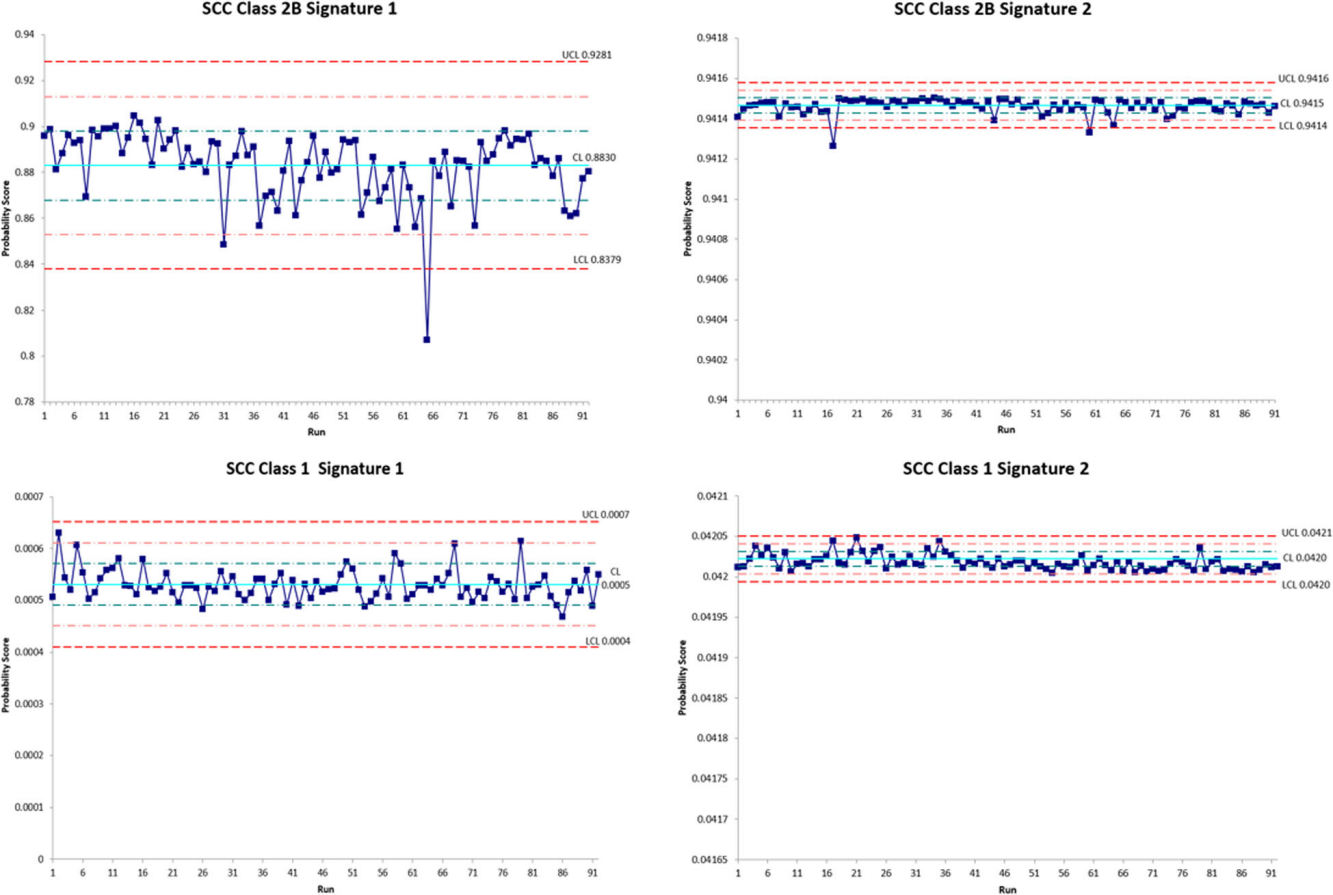
A Levey-Jennings graph depicts the positive control sample probability score stability and assay robustness across multiple runs. The reproducibility of the assay performance for a Class 1 and Class 2B positive control is shown. The mean is shown as the control limit (CL); +1SD is the dashed green line; +2SD is the dashed pink line; +3SD UCL (upper control limit) is the dashed red line; −3SD LCL (lower control limit) is the dashed red line

Control processes were implemented to ensure the accuracy and precision of the 40-GEP assay for clinical testing. Positive and negative controls were included with every batch of clinical samples, in addition to no-template, water controls and a qPCR DNase control to assess potential DNA contamination. The reproducibility of the assay performance was monitored with well-characterized, previously run, positive controls (Class 1 and Class 2B) to ensure all experiments described in the validation performed within clinically acceptable confidence limits (±2 standard deviations [SD]). The performance of the control’s mean probability scores was evaluated with Levey-Jennings analysis to illustrate the stability and assay robustness across multiple runs **(**Fig. 3**)**. Class 1, Signature 1 controls had a mean probability score of 0.00053 and a standard deviation (SD) of 0.00003, with a range of 0.00047–0.00063. Class 1, Signature 2 controls had a mean probability score of 0.042018 and a SD of 0.000009, with a range of 0.042004–0.042048. Class 2B, Signature 1 controls had a mean probability score of 0.883023 and a SD 0.014953, with a range of 0.806996–0.904568. Class 2B, Signature 2 controls had a mean probability score of 0.941408 and a SD of 0.000510, with a range of 0.936554–0.941503. These control samples were included from experiment to experiment and across multiple reagent lots. No assay was rejected due to amplification of a negative control or a failed positive control probability score. The control limit (CL), measured as the mean quantitative probability scores, upper control limit (UCL), and lower control limit (LCL) were also established by Levey-Jennings analysis. The Class 1, Signature 1 positive control sample had a CL of 0.0005, UCL of 0.007, and LCL of 0.004. The Class 1, Signature 2 positive control sample had a CL of 0.0420, UCL of 0.0421, and LCL of 0.0420. The Class 2B, Signature 1 positive control sample had a CL of 0.8830, UCL of 0.9281, and LCL of 0.8379. Lastly, the Class 2B, Signature 2 positive control sample had a CL of 0.9415, UCL of 0.9416, and LCL of 0.9414. These data support the robustness of the assay and reflect its ability to consistently perform to expectations.

### Reproducibility- inter- and intra-assay reliability, inter-operator precision

To assess the inter-assay reliability of the 40-GEP test, a precision experiment was performed by running 30 samples in duplicate (1 & 2) for the original run, with a subsequent run prepared in duplicate (3 & 4) on a different day with a different instrument and analyst **(**Table 1**)**. Controls were included for each run, as described in Methods. The class calls resulting from the average probability scores of both the Signature 1 and Signature 2 components of the algorithm were evaluated, followed by determination of class assignment concordance. One sample was removed due to dilution error, resulting in a total of 29 evaluable samples. Following class call analysis, 27/29 (93%) samples were determined to be concordant for the class call assignment. Of the two cases that changed class call, one class call change was from Class 2A to Class 1 and the other was from Class 2A to Class 2B. The bias analysis (mean absolute difference) in matched probability scores was − 0.04 for Signature 1 and 0.02 for Signature 2, demonstrating that the variability was within normal allowable clinical variation and not likely to change class assignment.

**Table 1:** Inter-assay summary data for replicates 1 and 2 vs 3 and 4 were used for the evaluation of Class call concordance. 30 FFPE available samples were run in duplicate, for a total of 4 replicates, over 2 days by two different technologists and using two different machines. The individual sample class call averages of the replicates are shown

	Avg Class Call for Replicates		
Sample	1&2	3&4	Concordant
1	Class 1	Class 1	Yes
2	Class 1	Class 1	Yes
3	Class 2A	Class 2A	Yes
4	Class 1	Class 1	Yes
5	Class 1	Class 1	Yes
6	Class 1	Class 1	Yes
7	Class 1	Class 1	Yes
8	Class 2A	Class 2A	Yes
9	Class 1	Class 1	Yes
10	Class 1	Class 1	Yes
11	Class 1	Class 1	Yes
12	Class 1	Class 1	Yes
13	Class 1	Class 1	Yes
14	Class 1	Class 1	Yes
15	Class 2A	Class 1	No*
16	Class 1	Class 1	Yes
17	Class 1	Class 1	Yes
18	Class 1	Class 1	Yes
19	Class 2A	Class 2A	Yes
20	Class 1	Class 1	Yes
21	Class 2A	Class 2A	Yes
22	Class 1	Class 1	Yes
23	Class2B	Class2B	Yes
24	Class 2A	Class2B	No*
25	Class 2A	Class 2A	Yes
26	Class2B	Class2B	Yes
27	Class 1	Class 1	Yes
28	Class 2A	Class 2A	Yes
29	Class2B	Class2B	Yes
30	Class2B	Class 2A	No**

*Not concordant

**Excluded, dilution error

Inter-operator precision was also assessed during the inter-assay experiment and was determined by the evaluation of 20 samples with two different operators on separate days on two different machines. Inter-machine precision was evaluated by use of the same 20 samples by the same two operators on two different QuantStudio 12 k Flex machines (ThermoFisher) on different days. The class call concordance was considered acceptable with 19/20 (95%) samples determined to be concordant (data on file).

For the intra-assay precision experiment, 46 FFPE samples were assayed **(**Table 2**)** with each sample run in duplicate (for a total of 4 replicates) by the same technologist for an open array (5 samples per array, with 10 arrays on a single instrument over 2 days) from one RNA extraction event of sample preparation. The class calls resulting from the average probability scores for both the Signature 1 and Signature 2 components of the algorithm of replicates 1 & 2 were compared to the class calls resulting from the average probability scores for both the Signature 1 and Signature 2 components of the algorithm for replicates 3 & 4. To be considered acceptable, the concordance needed to be ≥90% for class call. Following class call analysis, samples were concordant for class call assignment in 45/46 (98%) samples. The single case changing from Class 1 to Class 2A generated probability scores within 0.01 for the Signature 2 cut point (< 0.05), a difference that is within the SD of the reproducibility curves and expected clinical variability curves. The bias analysis in matched probability scores was − 0.03 for Signature 1 and 0.005 for Signature 2, showing variability to be within acceptable clinical variation and not likely to change class assignment.

**Table 2:** Intra-assay summary data for replicates 1 and 2 vs 3 and 4 were used for the evaluation of Class call concordance. 46 FFPE available samples were run in duplicate, for a total of 4 replicates, on the same day by the same technologist. The individual sample class call averages of the replicates are shown

	Avg Class Call for Replicates		
Sample	1&2	3&4	Concordant
1	Class 1	Class 1	Yes
2	Class 1	Class 1	Yes
3	Class 1	Class 1	Yes
4	Class 1	Class 1	Yes
5	Class 2A	Class 2A	Yes
6	Class 1	Class 1	Yes
7	Class 1	Class 1	Yes
8	Class 2A	Class 2A	Yes
9	Class 1	Class 2A	No*
10	Class 1	Class 1	Yes
11	Class 1	Class 1	Yes
12	Class 1	Class 1	Yes
13	Class 1	Class 1	Yes
14	Class 1	Class 1	Yes
15	Class 1	Class 1	Yes
16	Class 2A	Class 2A	Yes
17	Class 1	Class 1	Yes
18	Class 1	Class 1	Yes
19	Class 2A	Class 2A	Yes
20	Class 2A	Class 2A	Yes
21	Class 1	Class 1	Yes
22	Class 1	Class 1	Yes
23	Class 2A	Class 2A	Yes
24	Class 1	Class 1	Yes
25	Class 1	Class 1	Yes
26	Class 1	Class 1	Yes
27	Class 1	Class 1	Yes
28	Class 2A	Class 2A	Yes
29	Class 1	Class 1	Yes
30	Class 1	Class 1	Yes
31	Class 1	Class 1	Yes
32	Class 1	Class 1	Yes
33	Class 1	Class 1	Yes
34	Class 1	Class 1	Yes
35	Class 1	Class 1	Yes
36	Class 1	Class 1	Yes
37	Class 2A	Class 2A	Yes
38	Class 1	Class 1	Yes
39	Class2B	Class2B	Yes
40	Class2B	Class2B	Yes
41	Class 2A	Class 2A	Yes
42	Class 2A	Class 2A	Yes
43	Class2B	Class2B	Yes
44	Class 1	Class 1	Yes
45	Class 2A	Class 2A	Yes
46	Class2B	Class2B	Yes

*Not concordant

### Specimen and reagent stability

To assess RNA stability and longevity following long-term storage at − 80 °C, 23 FFPE samples were tested over a span of 146 days across multiple different runs **(**Table 3**)**. The class call concordance was 22/23 (96%) samples. To evaluate sample stability, specimens were assessed following uninterrupted processing of the assay versus following short-term storage. For short-term storage, the cDNA was held following reverse transcription (RT) (*n* = 15) at 4 °C for 96 h, prior to continuing through the entire assay. The samples were run on a single, open array with the same operator. The performance metrics and concordance analyses were deemed acceptable.

**Table 3:** Summary data for sample stability, longevity & reagent reproducibility. *RT = reverse transcription, OA = open array, MM = master mix*

Additional Characteristics	Sample	Overall Class Call Concordance	Acceptable
Longevity, RNA Stability	*n* = 23	96% (22/23)	≥ 90%
cDNA stability	*n* = 15 RT hold	100% (15/15)	≥ 90%
Lot to Lot Reagents Reproducibility	*n* = 10 Extraction	90% (9/10)	≥ 90%
	*n* = 11 RT Kit & RNase	91% (10/11)	≥ 90%
	*n* = 5 Pooled Assay & Pre-amplification MM	100% (5/5)	≥ 90%
	*n* = 8 OA & OA MM	100% (8/8)	≥ 90%

Lot-to-lot reproducibility for critical reagents was evaluated using between 5 and 11 samples over timespans ranging from 3 months (for assay reagents) to 11 months (for RNA extraction reagents), utilizing 2 to 3 different lots. Summary data from Table 3 illustrate a 90–100% class call concordance for all reagents: extraction reagents (9/10; 90%), RT Kit and RNase reagents (10/11; 91%), pooled assay and pre-amplification master mix reagents (5/5; 100%), and open array (OA) and open array master mix (OA MM) reagents (8/8; 100%). This class call analysis of the individual sample data from lot-to-lot shows consistent reproducibility.

### DecisionDx-SCC technical experience

**Fig. 4:**
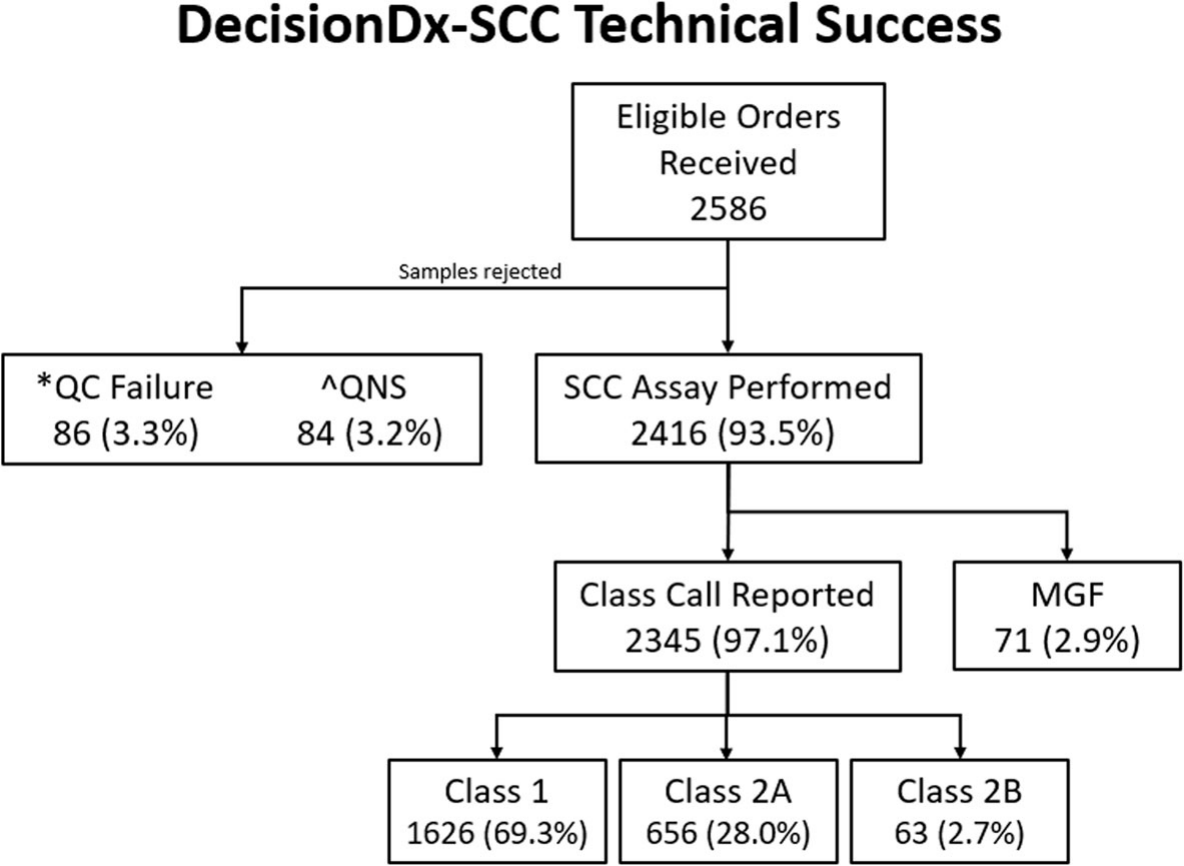
Technical experience of the DecisionDx-SCC test for orders submitted from September 1, 2020 – July 31, 2021. *QC failure = quality control failure (< 40% tumor content); ^QNS = quantity not sufficient (RNA < 5.0 ng/μl)

Over an 11-month time span, September 1, 2020 through July 31, 2021, 2586 eligible clinical orders for the 40-GEP test were received from across the United States **(**Fig. 4**)**. Of these, 86 (3.3%) and 84 (3.2%) samples were rejected due to QC failure (having an insufficient tumor content of < 40%) or “quantity not sufficient” (QNS; RNA < 5.0 ng/μl), respectively. Overall, 2345 (97.1%) of all tested samples gave successful, actionable class call outcomes (Class 1: 1626 (69.3%) samples, Class 2A: 656 (28.0%) samples, and Class 2B: 63 (2.7%) samples). The remaining 71 (2.9%) samples tested were reported as a MGF as the final result. The average turn-around time from receipt of tissue to report distribution was less than 5 days.

## Discussion

Effective molecular prognostic assays are becoming more predominant for clinical decision making of oncology patients. Due to this rapidly evolving field, it has become critical to provide reproducible and reliable risk assessment via analytical and clinical validity, along with demonstrations of added value to patient management decisions, defined as clinical utility [17]. Clinical validity and utility studies regarding the 40-GEP test not only support its ability to improve upon current methods of predicting a tumor’s metastatic risk, but also that physicians would appropriately integrate test results into patient management decisions [10, 11]. Here, we report the analytic validity of the assay by using accepted methods for reproducibility and technical reliability of clinical testing in accordance with published guidelines [14, 15].

Determination of minimum suitable RNA concentration necessary for accurate reproducibility of class call assignments is of the utmost importance. These data were achieved by following a comprehensive protocol previously published [9, 16]. Also, validation of the repeatability of the assay was shown using the Levey-Jennings quality control analysis which exhibited stringent upper and lower control limits. These robust results highlight the proficiency of the procedures used to perform the 40-GEP test in a centralized CLIA-certified, CAP-accredited, and New York State-approved laboratory.

The 40-GEP test is the first gene expression profile test for SCC. Thus, while the analytic framework for this type of test has been ascertained [16], the qualitative classification system of three possible risk groups is novel. The definitive precision of the assay can be seen by the 95% concordance in inter-operator precision, along with inter-assay and intra-assay reliability results which demonstrated 93 and 98% concordance for class call assignments, respectively. This confidence was also evident with the acceptable concordance of sample stability following long-term storage of RNA and short-term storage of cDNA following RT hold. Additionally, being that quality assurance for laboratory reagents are required as part of assay performance verification [18], we executed lot-to-lot reproducibility experiments for critical reagents with results meeting or exceeding the requirements for a clinically applied prognostic test [18].

Finally, an indication of the consistent high performance of the 40-GEP test can be seen by its technical success rate. Over an 11-month timeframe, 2586 40-GEP tests were ordered, of which 93.5% were eligible to be run on the assay. Of these tests, 97.1% were successful in giving valid class calls. The results provided by the 40-GEP test may help curb the dramatic increase seen in mortality of SCC patients after loss of locoregional control of disease by providing a patient with their individual risk of nodal or distant metastasis early in the disease state and informing appropriate management decisions based on risk.

## Conclusion

Following published guidelines for establishing analytic validity, the results of this study substantiate the accuracy and reliability of the DecisionDx-SCC test regarding its robust repeatability and reproducibility. The variability in sample and reagent stability was minimal, demonstrating the value of implementing the rigorous protocols used to perform the assay. The notable technical performance of the test also validated its strength as a robust clinical assay. In summary, this data, along with clinical validity and utility studies, show that the 40-GEP test can add value to high-risk SCC patient risk assessment with the potential to improve disease-related outcomes.

## Data Availability

Relevant data generated or analyzed during this study are included in this published article (and its supplementary information files). Additional data analyzed during the current study are available from the corresponding author on reasonable request.

## Abbreviations

40-GEP: 40-gene expression profile
CL: Control limit
FFPE: Formalin-fixed, paraffin embedded
H&E: Hematoxylin and eosin
IRB: Institutional Review Board
LCL: Lower control limit
MGF: Multiple gene failure
NCCN: National Comprehensive Cancer Network
OA: Open array
OA MM: Open array master mix
QC: Quality control
RT: Reverse transcription
SCC: Cutaneous squamous cell carcinoma
SD: Standard deviation
SOP: Standard operating procedure
UCL: Upper control limit
